# A panel of medaka isogenic lines suggests individual, seasonal, and sexual genetic variation of *bdnf* gene expression in the brain

**DOI:** 10.3389/fnbeh.2026.1681619

**Published:** 2026-04-22

**Authors:** Eleonora Rovegno, Christina Vasilopoulou, Saul Pierotti, Tomas Fitzgerald, Joachim Wittbrodt, Ewan Birney, Daniela Vallone, Felix Loosli, Cristiano Bertolucci, Nicholas S. Foulkes, Tyrone Lucon-Xiccato

**Affiliations:** 1 Department of Life Sciences and Biotechnology, University of Ferrara, Ferrara, Italy; 2 European Molecular Biology Laboratory, European Bioinformatics Institute, Wellcome Genome Campus, Hinxton, United Kingdom; 3 Centre for Organismal Studies, Heidelberg University, Heidelberg, Germany; 4 Institute of Biological and Chemical Systems - Biological Information Processing, Karlsruhe Institute of Technology, Eggenstein-Leopoldshafen, Germany

**Keywords:** genetic variation, individual differences, neurobiology, neurotrophins, *Oryzias latipes*

## Abstract

**Introduction:**

Once recognized only in humans, variance in the cognitive phenotype is now acknowledged in a range of vertebrate species. However, our understanding of its underlying causes is still incomplete. Brain-derived neurotrophic factor (BDNF) is an essential protein for brain functioning and plays a key role in cognitive processes such as learning and memory, including interindividual variation. Environmental factors influence BDNF abundance in the brain, and so do genetic polymorphisms in humans and mice.

**Methods:**

Using the Medaka Inbred Kiyosu-Karlsruhe (MIKK) panel of near-isogenic medaka lines, which captures a wide range of natural genetic variation in this species, we investigated the potential quantitative genetic variation in *bdnf* gene expression in the brain.

**Results:**

Our findings show significant variation in *bdnf* mRNA expression levels across MIKK lines, with a two-fold difference between the lines exhibiting lower and higher expression. Seasonal variation was also observed, with higher average *bdnf* levels in summer. However, a tentative analysis suggested that this average effect was not consistent across the lines, with some lines even showing significantly greater expression in winter. Similarly, across the entire sample, males and females did not differ in *bdnf* expression overall, although some lines displayed sex differences greater than expected by chance.

**Discussion:**

These results suggest that quantitative genetic differences, in concert with environmental influences, contribute to *bdnf* expression variability.

## Introduction

Individual cognitive differences in humans have been known and studied for more than a century (Apperly, 2012; Bolton, 1923; Carroll and Maxwell, 1979; Conway et al., 2021; Frischkorn et al., 2022; Gustafsson and Undheim, 1996). Growing evidence now suggests that other animals also display individual differences in cognitive traits such as learning, memory, and problem solving, with this variation observed across a wide range of taxa, from species closer to us, such as primates (Banerjee et al., 2009; Bohn et al., 2023), to other mammals (Galsworthy et al., 2005; Matzel and Sauce, 2017; Matzel et al., 2011; Thornton and Lukas, 2012), birds (Herrmann, 2016; Thornton and Lukas, 2012), and even one of the most ancient vertebrate groups, teleost fish (Lucon-Xiccato and Bisazza, 2017). Despite the increasing number of species studied, we still know relatively little about the underlying causes of cognitive phenotypic variance.

A protein from the neurotrophin family, BDNF, has attracted considerable attention as a potential molecular determinant of individual differences in cognition. BDNF evolved early in vertebrate history, before the divergence of cartilaginous fish (Hallböök et al., 1998), and it is present in all vertebrate groups that emerged thereafter, with a highly conserved structure and function (Götz et al., 1992). BDNF has broad activational and organizational effects on the vertebrate nervous system (Kowiański et al., 2018; Rose et al., 2004; von Bohlen und Halbach and von Bohlen und Halbach, 2018). Among these effects, *in vitro* studies on neurons have shown that BDNF plays a major role in synaptic plasticity (Lu et al., 2014; von Bohlen und Halbach and von Bohlen und Halbach, 2018; Zagrebelsky and Korte, 2014) and enables long-term potentiation (Alonso et al., 2002; Bekinschtein et al., 2008; Kovalchuk et al., 2002), which is considered to be the principal molecular mechanism underlying learning and memory formation (Cunha et al., 2010; Hall et al., 2000; Lynch, 2004; Yamada et al., 2002). Furthermore, *in vivo* evidence supports BDNF’s role in cognition. Mutant zebrafish lacking the *bdnf* gene display severe learning impairments, failing both simple visual discrimination and spatial learning tasks (Lucon-Xiccato et al., 2022b). Crucially, evidence also suggests a quantitative effect of BDNF on cognitive abilities. For example, post-mortem examination of BDNF expression levels has revealed a positive association with individual differences in cognitive abilities in humans (Buchman et al., 2016). In two recent studies on zebrafish, we also found that individuals’ levels of *bdnf* expression were highly variable and predicted learning performance more reliably than a panel of other genes involved in neural plasticity (Gatto et al., 2025; Lucon-Xiccato et al., 2022b).

To better understand the association between BDNF levels in the brain and individual differences in cognitive abilities, it is essential to understand the underlying mechanisms that result in variation in BDNF levels. A large body of evidence in mammals and fish shows that BDNF production varies according to an individual’s experiences (Branchi et al., 2006; Barros et al., 2019; Dandi et al., 2018; Kang et al., 2024; Mes et al., 2019, 2020; Nyman et al., 2017; Tognoli et al., 2010; Xu et al., 2021). For example, mice and rats raised in enriched environments produce more BDNF compared to those raised in barren environments (Novkovic et al., 2015) and fish that live in groups of different sizes express different levels of *bdnf* across various brain regions (La Loggia et al., 2025). Moreover, BDNF levels in humans are also modulated by epigenetic mechanisms (Boulle et al., 2012; D’Addario et al., 2013; Dell et al., 2014). Beside this environmentally driven plasticity, evidence suggests that BDNF levels may also be influenced at the genetic level. In humans, the Val66Met genetic polymorphism in the *BDNF* gene sequence does not affect the protein, but reduces its secretion (Egan et al., 2003), and this in turn determines cognitive impairment (Altmann et al., 2016; Forlenza et al., 2010; Guerini et al., 2009; Li Voti et al., 2011; Wang et al., 2019). However, the phenotypic variation provided by this single polymorphism does not seem sufficient to explain the large and continuous heritable variance in the cognitive phenotype (Croston et al., 2015; Galsworthy et al., 2005; Hopkins et al., 2014; Jansen et al., 2015; Langley et al., 2020; Pesta et al., 2020; Smith et al., 2015). Therefore, we hypothesize that there may be a more subtle quantitative genetic variation contributing to differences in BDNF levels and, consequently, to individual differences in the cognitive phenotype.

In this study, we used the Medaka Inbred Kiyosu-Karlsruhe (MIKK) panel of medaka (*Oryzias latipes*) lines (Fitzgerald et al., 2022) as a model to test the hypothesis of the influence of quantitative genetic variation on *bdnf* gene expression levels. We investigated the presence of differences in *bdnf* expression levels in the brain of medaka MIKK lines, which would suggest the presence of quantitative genetic variation. While outbred populations are often used for such studies, they typically require complex experimental designs (e.g., half-sib/full-sib) and very large sample sizes to achieve sufficient statistical power to partition genetic from environmental variance. Conversely, common laboratory strains often result from non-standardized inbreeding or undocumented hybridization from founders with unknown origins (e.g., local commercial sources), which may not accurately reflect natural genetic variability. The MIKK panel overcomes these limitations by providing a standardized genomic resource consisting of near-isogenic inbred medaka lines derived from a polymorphic wild founder population through single full-sibling-pair (brother-sister) crosses for 9 generations (Fitzgerald et al., 2022), thus representing a model of the genetic variation present in a wild population (Fitzgerald et al., 2022). As established by Fitzgerald et al. (2022), the spectrum of variation is predominantly intergenic and intronic. Given that non-coding regions comprise approximately 95% of the medaka genome, the quantitative variation in *bdnf* expression investigated in this study likely stems from regulatory differences rather than coding mutations. Furthermore, the near-isogenic structure of these lines is particularly well-suited for identifying polygenic influences on gene expression, as it allows for the reliable detection of complex phenotypic effects arising from multiple loci that would be difficult to isolate in outbred or non-standardized models. Taken together, this allows for the high-resolution study of natural variation within a controlled laboratory framework, a tool that is currently unavailable in other teleost models.

Given that previous research has reported that learning varies between summer and winter conditions and between males and females in medaka (López-Olmeda et al., 2021; Lucon-Xiccato et al., 2022a), we took advantage of our dataset to preliminarily investigate whether seasonal and sexual variation in *bdnf* expression levels exist in this species. Seasonal variation was assessed by comparing individuals maintained under two contrasting photoperiod regimes, reflecting the natural day/night cycles experienced by medaka in summer (14 h light: 10 h dark) and winter (10 h light: 14 h dark). In this species, photoperiod length serves as a primary environmental cue signaling seasonal change and triggering a suite of plastic adjustments in physiology, metabolism, growth, behavior, and cognition (Awaji and Hanyu, 1989; Davis et al., 2002; Fujisawa et al., 2021; Lucon-Xiccato et al., 2022a; Urasaki, 1976). The two light regimes therefore have opposing biological significance, as medaka reproduce exclusively under summer conditions and show regression of reproductive traits during winter (Awaji and Hanyu, 1988; Koger et al., 1999). Sexual variation was evaluated by analyzing *bdnf* expression in both male and female individuals.

## Materials and methods

### Fish maintenance

The medaka lines of the MIKK panel were maintained at the Institute of Biological and Chemical Systems – Biological Information Processing, Karlsruhe Institute of Technology (KIT). Care was taken to ensure that all subjects experienced identical rearing conditions to avoid environmental effects on bdnf expression. Individuals from each strain were housed in homogeneous social groups in separate 6 L tanks. Each tank was connected to an automatic water recirculation and filtration system that maintained constant standard conditions, including a temperature of 26°C (Loosli et al., 2000).

To study seasonal variation of *bdnf* expression, we kept one tank per each line under summer photoperiod conditions (14 h light: 10 h dark) and one tank under winter photoperiod conditions (14 h light: 10 h dark). This treatment was conducted in the same 6-L tanks and under the same maintenance conditions described above, including a fixed temperature of 26°C for both treatments. The lights were regulated using automated timers. To ensure that fish from the two conditions were not influenced by the lighting of the other treatment, the treatments were conducted in two separate rooms.

### Brain dissection and RNA sequencing

1-year-old adult medaka (*n* = 166 from 53 MIKK lines) were euthanized via hypothermic shock for brain dissection. After dissection, brains were immediately shock frozen in liquid nitrogen and then used for RNA extraction. A Qiagen automated extraction platform was used to extract RNA from the brain samples with QIAsymphony RNA Kits. We extracted polyA RNA for paired end RNA-Seq analysis. NEBNext Ultra II Directional RNA Library Prep Kit for Illumina was used to prepare samples for Illumina RNA sequencing. Samples were then sequenced on a Hiseq 4000 sequencing platform following the manufacturer’s instructions.

### RNA sequencing data processing

For the pre-processing, alignment and quantification of the RNA-Seq dataset, we used the bioinformatics pipeline nf-core/rnaseq v3.12.0 (Ewels et al., 2020; Patel et al., 2025). The samples were aligned against the medaka HdrR reference genome from ENSEMBL (release 103) using STAR v2.7.9a and were further processed for downstream BAM-level quantification with Salmon v1.10.1. After investigating the comprehensive MultiQC reports from the nf-core/rnaseq pipeline, we removed samples that failed the strandedness checks, samples with an unusual distribution of GC content, and samples with less than 50% of uniquely mapped reads. To identify and remove tissue outliers based on median absolute deviation, we used nf-core/differentialabundance v1.4.0 nextflow pipeline (Wacker et al., 2023; Ewels et al., 2020). We further explored the presence of potential sample swaps and mislabeling by developing a SNP check pipeline that assesses whether the assigned MIKK line for each sample is the best match based on calculated genotype proportions from BAM read counts. For the SNP check pipeline, we selected 1377 high-confidence SNPs passing the following criteria: only high-coverage SNPs, SNPs mapped in exons and not located in chromosome 2. The genotype ratios were calculated for all 53 MIKK lines, based on the ratio of SNPs that match the expected genotype. For every sample, we compared the assigned MIKK line’s proportion of matching SNPs to the most compatible line’s proportion. Samples were retained only if the difference in matching-SNP proportion between the assigned line and the most compatible line exceeded a strict threshold (difference > −0.005), ensuring that the assigned line provided the best-supported genetic match. A total of 131 samples from 36 MIKK lines passed the selection criteria. For this study, we considered only MIKK lines that had one data point per condition (female summer, female winter, male summer, male winter), for a total of 25 lines and 100 samples analyzed.

### Statistical analyses

Analyses were performed in RStudio version 2024.12.1+563. To determine whether *bdnf* expression varies among medaka lines, which would suggest underlying genetic variance, we compared two models differing in the inclusion of line as a random effect. Both models were fitted with *bdnf* expression as the dependent variable, and with sex and season as fixed effects. The interaction between sex and season was removed due to the lack of significance (*P* > 0.5). The model without the random effect was a linear model fitted with the “*lm*” function. The model with the random effect was a linear mixed-effects model fitted with the “*lmer*” function from the “*lme4*” package. We estimated the total variance explained by each model: for the linear model, we used the multiple R^2^; for the linear mixed-effect model, we used the conditional R^2^, which includes both fixed and random effects. For the linear mixed-effect model, we also calculated the variance explained by the random effect using the intraclass correlation coefficient (Galwey, 2014). Last, for both models, we calculated the Akaike information criterion (AIC), which provides an indication of the goodness of the model fit (with lower AIC values indicating a better-fitting model and higher values suggesting a poorer fit) (Burnham et al., 2011).

To assess the average effect of season and sex in the population, we checked the significance of the terms in the linear mixed-effects model with the “Anova” function of the library “car.” To assess the variability of seasonal and sexual expression of *bdnf* among medaka lines we used a bootstrapping approach. First, we calculated percentage indexes of variation between seasons and between sexes. These were obtained as (average summer values for the line − average winter values for the line)/(average summer values for the line) and as (average female values for the line − average male values for the line)/(average female values for the line). Note that these indexes are based on examining a limited number of subjects per level, for example, two individuals per season per line and two individuals per sex per line. This design was adopted since, given the isogenic nature of the medaka lines, increasing the number of subjects per level would not increase the number of biological replicates. While allowing this type of analysis with a low sample size represents one of the strengths of the medaka panel, we prefer, out of caution, to refer to these results as exploratory and preliminary. We then generated 1000 bootstrap samples by resampling the two indexes and estimated 95% confidence intervals (CIs) for the mean. The mean and confidence intervals obtained via bootstrapping were not added to the observed data but used as thresholds for comparison with the observed data. Medaka lines outside the CI range were considered statistically deviant from the population mean.

## Results

### Variation of *bdnf* expression between lines

**FIGURE 1:**
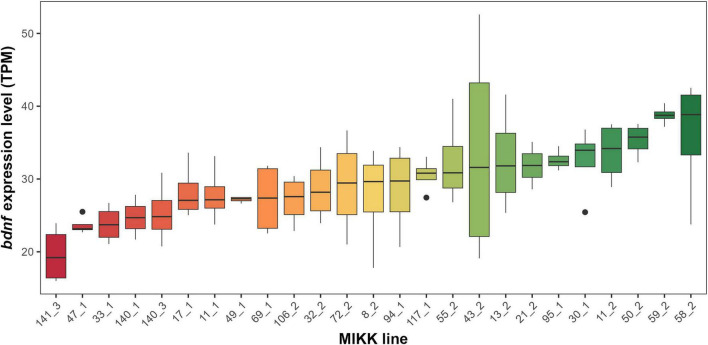
Average *bdnf* expression (Transcripts Per Million, TPM) variability across different Medaka Inbred Kiyosu-Karlsruhe (MIKK) lines. The boxes represent the interquartile range, the horizontal line within the box indicates the median, and the whiskers extend to 1.5 times the interquartile range. Lines are ordered on the *x* axis by the median *bdnf* expression. Outliers are shown as individual points.

The medaka lines showed significant variation in their average expression of *bdnf* (Figure 1). The difference between the medaka lines with lowest and highest values of *bdnf* expression was approximately two-fold (98.04%).

In the linear mixed-effects model, the random effect of the medaka line explained 31.20% of the variance in *bdnf* expression, for a total variance explained by the model of 34.00% (Table 1). Conversely, the model without the random effect only explained 4.06% of the variance in *bdnf* expression. The AIC (Table 1) and the statistical test (LRT_1_ = 11.156, *P* < 0.001) both confirmed that the model with the medaka line as the random effect explained significantly more variance in *bdnf* expression.

**TABLE 1:** Comparison between the fitting of the models with and without the medaka line as the random effect.

Model	Random effect	R^2^	AIC
Linear model	None	0.04	658.90
Linear mixed-effects model	Medaka line	0.34	644.21

The dependent variable was the expression levels of *bdnf*; the models were fitted with season and sex as fixed effects; R^2^ was multiple for the linear model and conditional for the linear mixed-effects model.

### Seasonal variation

**FIGURE 2:**
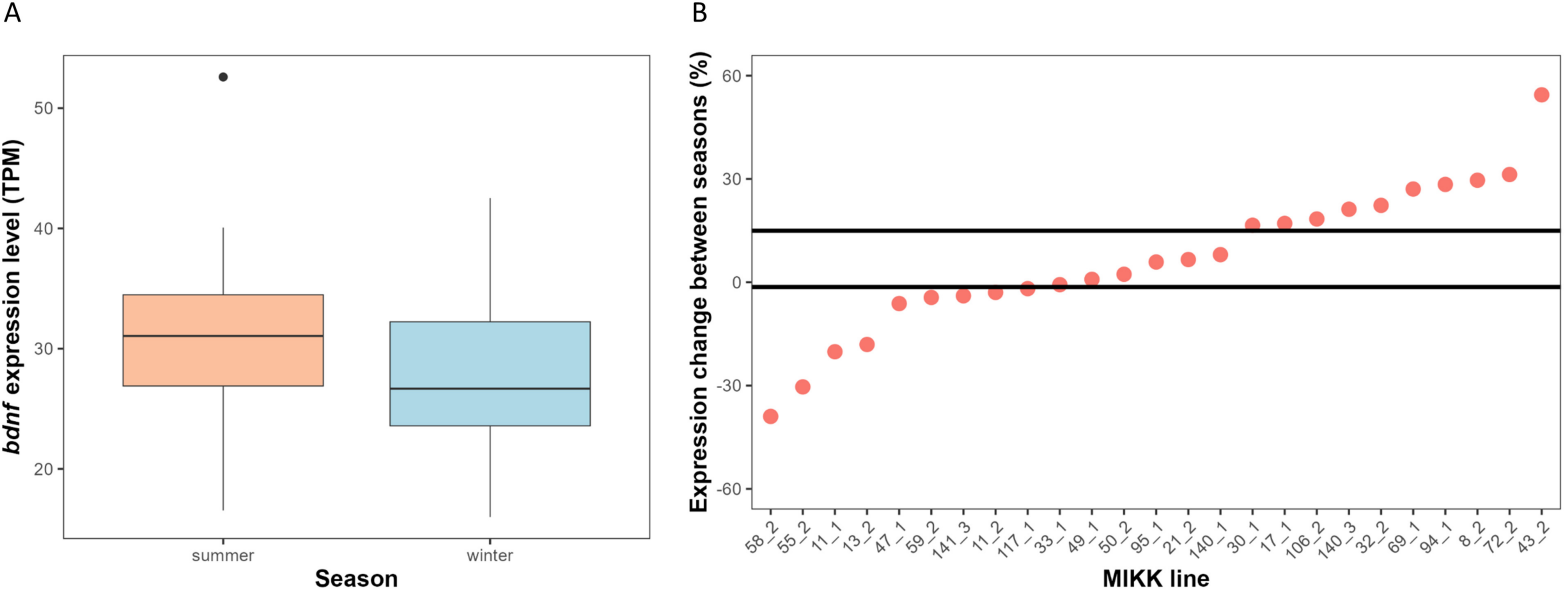
Seasonality of *bdnf* expression. **(A)** Boxplot comparing the average expression of *bdnf* between summer and winter conditions. Outliers are shown as individual points. **(B)** Divergence from chance of the percentage change in *bdnf* expression between seasons across the MIKK lines. Dots indicate the percentage change of each medaka line; positive values of the dots indicate greater expression during summer condition and negative values greater expression during winter condition. Black lines indicated 95% CI obtained with bootstrapping method.

The linear mixed-effects model revealed a significant effect of season (X^2^_1_ = 5.946, *P* = 0.015). Under summer photoperiod conditions, the average expression levels of *bdnf* were higher as compared to under winter photoperiod conditions (Figure 2A). Despite this general trend, there was substantial variation among lines in the seasonal expression of *bdnf*: the percentual change between summer and winter across the different lines ranged from −38.96% to 54.43%. The exploratory bootstrap analysis indicated that the seasonal change in *bdnf* expression of 17 out of 25 lines significantly differed from chance level (Figure 2B). Notably, 4 out of these 17 lines showing significant divergence expressed more *bdnf* under winter conditions (Figure 2B).

### Sexual variation

**FIGURE 3:**
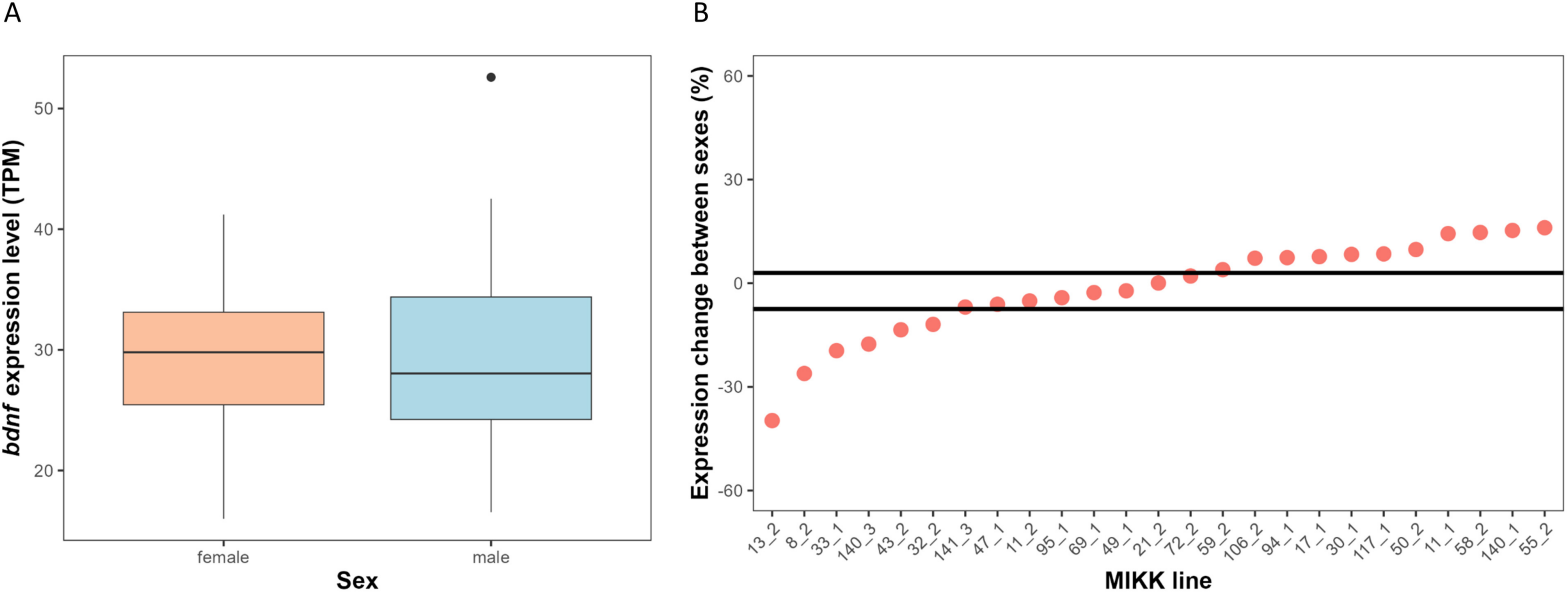
Influence of sex on *bdnf* expression. **(A)** Bar plot comparing the average expression of *bdnf* between males and females medaka. Outliers are represented by black dots. **(B)** Divergence from chance of the percentage change in *bdnf* expression between sexes across the MIKK lines. See Figure 2B caption for further explanation.

The effect of sex was not significant (X^2^_1_ = 0.009, *P* = 0.924), suggesting average similar levels of *bdnf* expression in males and females in the entire population (Figure 3A). However, the exploratory bootstrap analysis of the difference in expression between males and females within line showed significant variation: in 10 out of 25 lines, females expressed more *bdnf* than expected by chance; in 6 out of 25 lines males expressed more *bdnf* than expected by chance (Figure 3B).

## Discussion

Our analyses revealed that the MIKK panel of near-isogenic medaka lines displays a continuum of differences in *bdnf* expression. Indeed, the contribution of the line random effect to the variance explained by our model was significant. This suggests that variation in *bdnf* expression within a population, and hence BDNF levels in the brain, might arise, at least in part, from quantitative genetic differences.

While the significant differences in *bdnf* expression observed across the MIKK panel point toward substantial genetic control, it is important to note that these differences represent broad-sense genetic variance. Because the lines are near-isogenic and have been maintained independently for multiple generations, the phenotypic variation we report likely reflects complex polygenic interactions. Line-specific epigenetic contributions are also difficult to rule out, although the standardized husbandry and the consistent number of generations across lines are expected to minimize environmental effects.

Nevertheless, the finding that quantitative genetic differences contribute to the variation of brain BDNF levels has important consequences for our understanding of cognitive individualities. Variation in *bdnf* expression has been associated with large individual differences in cognition (Gatto et al., 2025; Lucon-Xiccato et al., 2022b), raising the question that motivated the present study: What mechanisms generate continuous variation in *bdnf* expression? As our results suggest that a component of the total variance in *bdnf* expression is genetic, this indirectly implies that an individual’s cognitive abilities may be, at least in part, influenced by this genetic component. Our conclusion, if confirmed by future studies, could explain the growing body of evidence in different animal species indicating heritability of cognitive skills (Bisazza et al., 2000; Galsworthy et al., 2005; Hopkins et al., 2014; Langley et al., 2020; Smith et al., 2015; Croston et al., 2015; Vila-Pouca et al., 2022). It is important to note that the genetic variation detected in our study is continuous (Figure 1) and thus offers a more likely explanation for the broad range of cognitive phenotypes observed as compared to the discrete variation associated with single polymorphisms described previously. We acknowledge that using whole-brain expression analysis may partially obscure the finer, region-specific relationships between BDNF and cognition. However, previous research in other fish models, such as zebrafish, has demonstrated that whole-brain *bdnf* levels can serve as a reliable predictor of overall cognitive performance (Gatto et al., 2025; Lucon-Xiccato et al., 2022b).

Genetic variation in *bdnf* expression is certainly not the only determinant of cognitive individualities. Indeed, studies in various animal models have shown that *bdnf* expression is influenced by an individual’s experience of the environment (Barros et al., 2019; Dandi et al., 2018; Kang et al., 2024; Mes et al., 2019; Tognoli et al., 2010). Therefore, we anticipate that the impact of BDNF on cognitive abilities is twofold, simultaneously determined by both genetic factors and experience. Furthermore, other proteins may play roles as important as BDNF given that cognitive traits are likely quantitative and controlled by numerous other genes (Hindley et al., 2023; Kovas and Plomin, 2006; Le Hellard and Steen, 2014). Overall, this is expected to further contribute to the array of individual differences observed in cognition.

Our analysis across all the lines also suggests that *bdnf* may undergo expression changes related to photoperiod conditions. On average, *bdnf* expression was higher during summer and lower during winter photoperiod conditions. While our findings specifically represent photoperiod-dependent regulation, photoperiod is known to induce seasonal plasticity in medaka (Lucon-Xiccato et al., 2022a). We therefore interpret our findings as possible evidence of seasonal variation in *bdnf* expression in medaka, as photoperiod was used as a primary driver to simulate seasonal transitions. Seasonal variation in BDNF has been previously detected in a study on humans’ serum (Molendijk et al., 2012) and in a study on mice brain (Adonina et al., 2024). Interestingly, two studies have shown that photoperiod treatment influences the cognitive phenotype of medaka (López-Olmeda et al., 2021; Lucon-Xiccato et al., 2022a). For example, males exhibited reduced learning ability under summer conditions (López-Olmeda et al., 2021). It is therefore possible that photoperiod-mediated regulation of *bdnf* expression contributes to cognitive plasticity across seasons. However, the temporal dynamics of this regulation require further investigation. While it remains unclear how rapidly these changes occur, a conditioning of 30 days ensures full acclimatization (López-Olmeda et al., 2021) and photoperiod-induced physiological states persist as long as the environmental cue remains. Mechanistically, this variation likely integrates into a broader seasonal transcriptomic program. In medaka, thousands of genes in the hypothalamus and pituitary oscillate seasonally, including pituitary hormones and transcription factors (Nakayama et al., 2023). Notably, the seasonal oscillation of genes involved in cell proliferation and differentiation suggests that *bdnf* variation may be a key factor in the neurobiological pathways governing seasonal brain remodeling and the regulation of neurogenesis.

Related to seasonality, we detected a second potential effect. While, on average, the entire medaka sample had higher *bdnf* transcript levels in summer, some isogenic lines displayed the opposite pattern, with higher *bdnf* levels during winter conditions. This effect should be interpreted with caution, as it was revealed based on comparisons within lines, under the assumption that each line, being nearly isogenic, represents a single biological replicate. However, according to our analyses, the effect is unlikely to be due to chance. This result suggests that seasonal variation in BDNF may not be universal within a population and that different individuals may exhibit distinct seasonal fluctuations. The underlying reason for this variation remains unclear. We suggest that future studies should investigate the adaptive consequences of producing different levels of BDNF during winter across environments to better understand this phenomenon.

The last factor we investigated, the sex, yielded complex results. First, there were no average differences in *bdnf* transcript levels between males and females. This was unexpected, as fish often exhibit sex-specific differences in cognitive abilities (reviewed in Lucon-Xiccato, 2022), and such differences have also been reported in medaka (López-Olmeda et al., 2021). We speculate that factors such as sex hormones could play a more prominent role than BDNF in shaping these sex differences in cognition (Gurvich et al., 2018; Healy et al., 2010; Ter Horst et al., 2012). Despite the absence of sex differences in the overall population, we found significant variation at the isogenic line level. While this should be interpreted cautiously given the aforementioned limitations of the panel-based approach, the data suggest that in some medaka lines males had higher BDNF levels, whereas in other lines, females did. As observed with seasonal variation, this suggests that genetic factors may determine whether males or females have higher *bdnf* expression. It could be explained by sex-specific selection pressures in different medaka populations. This, along with other ecological explanations, could be explored through surveys comparing medaka from different habitats.

In conclusion, our study has demonstrated the presence of a quantitative genetic component in individual, seasonal, and sex-related variation in *bdnf* gene expression within the medaka brain. These findings raise several questions for future research. How do genes and the environment interact to regulate *bdnf* expression in the brain? Do BDNF receptors also vary among individuals, potentially providing an additional explanation for cognitive variation that warrants further investigation? Does individual variation also occur during development, leading to organizational effects on the nervous system? Which genomic variants are responsible for BDNF levels differences across genotypes? Given the importance of BDNF in shaping cognitive phenotypes (Gatto et al., 2025; Lucon-Xiccato et al., 2022b), it is crucial to extend our investigations into its variation, including the use of alternative models such as the MIKK panel.

## Data Availability

The data that support the findings of this study are available on request from the first author. Requests to access these datasets should be directed to ER, rvglnr@unife.it.
